# Weekend effect on mortality by medical specialty in six secondary hospitals in the Helsinki metropolitan area over a 14-year period

**DOI:** 10.1186/s12913-020-05142-4

**Published:** 2020-04-17

**Authors:** Morag Tolvi, Kimmo Mattila, Jari Haukka, Leena-Maija Aaltonen, Lasse Lehtonen

**Affiliations:** 1 Department of Otorhinolaryngology – Head and Neck Surgery, University of Helsinki and Helsinki University Hospital, P.O. Box 263, 00029 HUS, Helsinki, Finland; 2 Group Administration, University of Helsinki and Helsinki University Hospital, Helsinki, Finland; 3 Clinicum, Department of Public Health, University of Helsinki, Helsinki and Faculty of Medicine and Health Technology, University of Tampere, Tampere, Finland; 4 Diagnostic Center, Helsinki University Hospital and University of Helsinki, Helsinki, Finland

**Keywords:** Hospital mortality, Patient discharge, Treatment outcome, Weekend effect, Quality of healthcare

## Abstract

**Background:**

The weekend effect is the phenomenon of a patient’s day of admission affecting their risk for mortality. Our study reviews the situation at six secondary hospitals in the greater Helsinki area over a 14-year period by specialty, in order to examine the effect of centralization of services on the weekend effect.

**Methods:**

Of the 28,591,840 patient visits from the years 2000–2013 in our hospital district, we extracted in-patients treated only in secondary hospitals who died during their hospital stay or within 30 days of discharge. We categorized patients based on the type of each admission, namely elective versus emergency, and according to the specialty of their clinical service provider and main diagnosis.

**Results:**

A total of 456,676 in-patients (292,399 emergency in-patients) were included in the study, with 17,231 deaths in-hospital or within 30 days of discharge. A statistically significant weekend effect was observed for in-hospital and 30-day post-discharge mortality among emergency patients for 1 of 7 specialties. For elective patients, a statistically significant weekend effect was visible in in-hospital mortality for 4 of 8 specialties and in 30-day post-discharge mortality for 3 of 8 specialties. Surgery, internal medicine, and gynecology and obstetrics were most susceptible to this phenomenon.

**Conclusions:**

A weekend effect was present for the majority of specialties for elective patients, indicating a need for guidelines for these admissions. More disease-specific research is necessary to find the diagnoses, which suffer most from the weekend effect and adjust staffing accordingly.

## Background

The weekend effect is the phenomenon of a patient’s day of admission affecting their risk for mortality [1]. An end-of-week effect, i.e. a higher risk of mortality on Friday, Saturday, Sunday or Monday, has also been documented [2–5]. Much debate surrounds the suspected reasons behind the weekend effect: less elective patients at the weekend, [6] sicker patients at the weekend [7] and the unreliability of administrative data in regard to the variability in coding practices and the lack of certain information, e.g. co-morbidities and disease severity [8–12]. Many disease-specific studies have been carried out with conflicting results [13–18]. At the forefront of weekend effect research have been the United States, Great Britain and Australia with only a few disease-specific studies in the Nordic countries [17, 19–22]. Our study brings a specialty-specific point of view with one of the largest weekend effect databases in the Nordic countries. By examining specialty-specific mortality, we can distinguish which specialties would benefit most from changes in procedure and staff, allowing for prudent division of resources.

The Helsinki Hospital District numbers 23 hospitals with a catchment area of approximately 1.6 million inhabitants and its secondary hospitals cover approximately 400,000 inhabitants [23]. In this study, we examine the six secondary hospitals of the hospital district in an attempt to delve into the effects of the centralization of services on mortality. In our study focusing on the university hospital of the hospital district, we found a weekend effect in in-hospital and 30-day post-discharge mortality for almost all non-centralized specialties and half of centralized specialties amongst elective patients. About half of centralized specialties had a weekend effect in in-hospital mortality amongst emergency patients [24].

## Methods

Of the administrative data of 28,591,840 patient visits from the years 2000–2013 in our hospital district, we extracted those in-patients treated in secondary hospitals who died during their hospital stay or within 30 days of discharge. We examined only those patients treated solely in secondary hospitals in order to investigate whether there was still a weekend effect after centralization of certain severe conditions to the university hospital. We eliminated those whose records were missing data, as well as day surgery patients and those admitted and discharged on the same day. Patients were then categorized according to the urgency of admission (elective versus emergency) and the specialty of their clinical service provider and main, most costly diagnosis, e.g. if a total hip replacement patient died of pneumonia, they were classified as a surgical patient. These specialties numbered eight: acute psychiatry, surgery, gynecology and obstetrics, internal medicine, pulmonology, neurology, pediatrics and otorhinolaryngology. The treatment of emergency otorhinolaryngology patients is centralized to the university hospital. Therefore, only elective patients were included in this data.

The study was reviewed and approved by the Research Administration of the Helsinki and Uusimaa Hospital District (Y1014KORV1). Ethics committee approval was not necessary due to national legislation which does not require ethics committee approval for a retrospective registry study without patient intervention, in accordance with the Medical Research Act of Finland [25, 26]. The datasets used and analyzed during the current study are available from the corresponding author on reasonable request.

### Outcomes

In-hospital mortality and 30-day post-discharge all-cause mortality were investigated in this study. We focused on examining the weekend effect and also identifying whether an end-of-week effect exists. We defined the weekend effect as higher mortality for patients admitted between midnight of Friday night and midnight of Sunday night and the end-of-week effect as higher mortality for patients admitted between midnight of Thursday night and midnight of Monday night. Public holidays occurring during the week are not included as weekends. These numbered seven to ten per year for the years of the study. This small number was unlikely to affect our findings radically and this same approach has been applied previously [11].

### Covariates included in the study

Covariates examined in the study included age by age group, sex (male or female), admission day of the week (Sunday through Saturday), admission month (January through December), admission year (2000 through 2013), urgency of admission (elective or emergency), specialty of clinical service provider and risk category. Age was categorized by age group: < 20, 20–39, 40–49, 50–59, 60–69 and 70+ years old. Pediatric patients were grouped as follows: < 12 months, 12–23 months, 2 years to 4 years 11 months, 5 years to 9 years 11 months, 10 years to 14 years 11 months, 15 years to 19 years 11 months and 20+ years old. In Finland, the cut-off age for pediatric patients is usually 16 years. However, if treatment is near completion when the patient turns 16, the pediatric department routinely finishes it off. Hence, there are patients over the age of 16 in the pediatric data.

### Statistical analysis

Due to a lack of information on co-morbidities and illness severity, we used five risk categories, into which we divided patients in this study according to the crude 30-day mortality rate by main discharge diagnoses (International Classification of Diseases, ICD-10) of the patients in this study [4]. A multivariable logistic regression model containing age, sex, risk category, weekday, year and month was applied to calculate the adjusted odds ratios (OR) with 95% confidence intervals (CI) using R language (R Core Team. R: A Language and Environment for Statistical Computing, Vienna, Austria: R Foundation for Statistical Computing 2019. https://www.R-project.org/).

## Results

### Deaths

A total of 456,676 in-patients (292,399 emergency in-patients) were included in the study, with 17,231 deaths in-hospital or within 30 days of discharge (Table 1), for an overall crude mortality rate of 3.8%. Emergency patients comprised 14,973 deaths (86.9%) for a crude emergency mortality rate of 5.1% and a crude elective mortality rate of 1.4%. The majority of deaths occurred in the age group of 70+ years for all specialties, except acute psychiatry (50–59 years old) and pediatrics (0–1 years old).

**Table 1:** Patient demographics of 456,676 in-patients between 2000 and 2013

	Acute Psychiatry	Surgery	Otorhinolaryngology	Gynecology & Obstetrics	Internal Medicine	Pulmonology	Neurology		Pediatrics
Total patients	26,664	151,063	7664	79,889	126,218	29,228	12,966		22,984
Total deaths	104	4220	5	181	9591	2487	617		26
Crude mortality rate of specialty (%)	0.4	2.8	0.1	0.2	7.6	8.5	4.8		0.1
Male deaths (% of deaths)	81 (77.9)	2196 (52.0)	3 (60)	0 (0)	4777 (49.8)	1621 (65.2)	287 (46.5)		12 (46.2)
Deaths in age group (n)									
< 20 years old (% of all deaths)	0	11 (0.3)	0 (0)	0 (0)	25 (0.3)	3 (0.1)	0 (0)	**0–1 years old**	11 (42.3)
20–39 (%)	19 (18.3)	32 (0.8)	0 (0)	6 (3.3)	83 (0.9)	13 (0.5)	5 (0.8)	**1–2**	4 (15.4)
40–49 (%)	21 (20.2)	136 (3.2)	0 (0)	12 (6.6)	212 (2.2)	47 (1.9)	9 (1.5)	**2–5**	3 (11.5)
50–59 (%)	28 (26.9)	365 (8.6)	0 (0)	44 (24.3)	693 (7.2)	251 (10.1)	36 (5.8)	**5–10**	4 (15.4)
60–69 (%)	17 (16.3)	719 (17.0)	0 (0)	40 (22.1)	1294 (13.5)	650 (26.1)	91 (14.7)	**10–15**	3 (11.5)
70+ (%)	19 (18.3)	2957 (70.1)	5 (100)	79 (43.6)	7284 (75.9)	1523 (61.2)	476 (77.1)	**15–20**	1 (3.8)
								**> 20**	0 (0)
Emergency deaths (%)	81 (77.9)	3562 (84.4)	3 (60)	127 (70.2)	8574 (89.4)	2070 (83.2)	539 (87.4)		17 (65.4)

### Weekend admissions

Weekend admissions, i.e. admissions occurring on Saturday or Sunday, numbered 17.8% (*n* = 81,277), encompassing 15.8% of acute psychiatry patients, 15.1% of surgery patients, 18.5% of gynecology and obstetrics patients, 21.1% of internal medicine patients, 17.8% of pulmonology patients, 20.5% of neurology patients, 21.6% of pediatrics patients and 0.4% of otorhinolaryngology patients.

### Emergency admissions

A statistically significant weekend effect was present amongst emergency patients in in-hospital mortality for internal medicine patients (*p* = 0.0000) (Table 2). In 30-day post-discharge mortality, a significant weekend effect was visible amongst internal medicine patients (*p* = 0.0001), with an end-of-the-week effect amongst surgery patients (*p* = 0.0364) (Table 3).

**Table 2:** Adjusted odds of in-hospital mortality in 292,399 emergency admissions and 164,277 elective admissions

SPECIALTY	Monday	Tuesday	Wednesday	Thursday	Friday	Saturday	Sunday
Acute psychiatry							
emergency	1.014 (0.141 to 7.268)	1.580 (0.261 to 9.561)	1	3.183 (0.634 to 15.977)	0.522 (0.047 to 5.824)	2.567 (0.423 to 15.595)	1.631 (0.227 to 11.747)
elective	0.255 (0.021 to 3.160)	2.015 (0.320 to 12.707)	1	0.000 (0.000 to Inf)	2.544 (0.371 to 17.464)	0.000 (0.000 to Inf)	0.000 (0.000 to Inf)
Surgery							
emergency	1.102 (0.911 to 1.334)	0.949 (0.779 to 1.156)	1	0.988 (0.809 to 1.206)	1.097 (0.899 to 1.338)	0.981 (0.792 to 1.215)	1.158 (0.942 to 1.424)
elective	0.946 (0.611 to 1.463)	0.748 (0.477 to 1.175)	1	0.837 (0.522 to 1.343)	1.466 (0.915 to 2.350)	3.153 (1.586 to 6.266)	1.095 (0.641 to 1.868)
Gynecology & obstetrics							
emergency	3.583 (0.707 to 18.164)	6.761 (1.347 to 33.931)	1	3.758 (0.716 to 19.714)	3.787 (0.725 to 19.783)	4.072 (0.737 to 22.508)	4.579 (0.830 to 25.255)
elective	4.939 (0.561 to 43.479)	3.741 (0.403 to 34.732)	1	1.449 (0.088 to 23.982)	5.149 (0.437 to 60.607)	21.255 (1.738 to 259.903)	7.869 (0.420 to 147.397)
Internal medicine							
emergency	1.137 (1.015 to 1.273)	1.133 (1.011 to 1.269)	1	1.089 (0.969 to 1.223)	1.135 (1.012 to 1.274)	1.230 (1.090 to 1.389)	1.380 (1.226 to 1.555)
elective	0.837 (0.601 to 1.167)	1.039 (0.757 to 1.425)	1	1.074 (0.765 to 1.507)	1.864 (1.353 to 2.568)	2.460 (1.711 to 3.537)	1.206 (0.801 to 1.815)
Pulmonology							
emergency	0.892 (0.705 to 1.129)	1.005 (0.793 to 1.273)	1	0.991 (0.781 to 1.259)	1.093 (0.862 to 1.387)	1.048 (0.812 to 1.354)	1.224 (0.954 to 1.572)
elective	0.886 (0.512 to 1.535)	0.996 (0.590 to 1.681)	1	1.329 (0.785 to 2.250)	1.897 (1.075 to 3.346)	2.632 (1.194 to 5.802)	2.642 (1.298 to 5.379)
Neurology							
emergency	1.241 (0.772 to 1.996)	0.893 (0.535 to 1.491)	1	1.263 (0.779 to 2.047)	1.464 (0.909 to 2.358)	1.373 (0.836 to 2.257)	1.509 (0.923 to 2.466)
elective	1.927 (0.598 to 6.209)	0.988 (0.301 to 3.244)	1	2.157 (0.671 to 6.937)	1.463 (0.367 to 5.830)	1.235 (0.278 to 5.493)	1.483 (0.325 to 6.772)
Otorhinolaryngology							
elective	0.000 (0 to Inf)	0.000 (0 to Inf)	1	12.892 (0 to Inf)	0.824 (0 to Inf)	1.994e+ 14 (0 to Inf)	0.275 (0 to Inf)
Pediatrics							
emergency	0.842 (0.042 to 16.711)	2.960 (0.235 to 37.299)	1	0.849 (0.044 to 16.441)	2.375 (0.182 to 31.073)	0.000 (0.000 to Inf)	0.996 (0.054 to 18.516)
elective	0.000 (0.000 to Inf)	0.969 (0.040 to 23.587)	1	0.000 (0.000 to Inf)	2.237 (0.064 to 78.594)	9.333 (0.269 to 324.341)	0.000 (0.000 to Inf)

**Table 3:** Adjusted odds of 30-day post-discharge mortality in 292,399 emergency admissions and 164,277 elective admissions

SPECIALTY	Monday	Tuesday	Wednesday	Thursday	Friday	Saturday	Sunday
Acute psychiatry							
emergency	1.394 (0.577 to 3.370)	1.164 (0.454 to 2.984)	1	0.884 (0.324 to 2.414)	1.483 (0.610 to 3.605)	1.086 (0.375 to 3.147)	1.513 (0.552 to 4.148)
elective	0.282 (0.045 to 1.768)	1.073 (0.223 to 5.165)	1	0.952 (0.150 to 6.032)	0.515 (0.052 to 5.130)	1.784 (0.167 to 19.113)	0.000 (0.000 to Inf)
Surgery							
emergency	1.180 (1.011 to 1.378)	0.978 (0.832 to 1.149)	1	1.056 (0.899 to 1.242)	1.010 (0.855 to 1.192)	1.000 (0.840 to 1.191)	0.975 (0.817 to 1.164)
elective	1.168 (0.866 to 1.576)	1.028 (0.759 to 1.392)	1	1.138 (0.830 to 1.559)	1.189 (0.819 to 1.727)	2.315 (1.354 to 3.958)	1.030 (0.702 to 1.511)
Gynecology & obstetrics							
emergency	0.758 (0.342 to 1.684)	0.338 (0.122 to 0.941)	1	1.093 (0.481 to 2.485)	0.786 (0.334 to 1.852)	1.134 (0.471 to 2.732)	1.258 (0.543 to 2.914)
elective	2.291 (0.622 to 8.443)	1.437 (0.351 to 5.885)	1	4.003 (1.053 to 15.220)	3.475 (0.663 to 18.210)	27.472 (4.218 to 178.921)	3.574 (0.335 to 38.115)
Internal medicine							
emergency	1.171 (1.044 to 1.314)	1.066 (0.947 to 1.200)	1	1.083 (0.960 to 1.220)	1.136 (1.009 to 1.279)	1.163 (1.026 to 1.320)	1.291 (1.141 to 1.461)
elective	1.311 (0.987 to 1.742)	1.074 (0.797 to 1.447)	1	1.262 (0.925 to 1.723)	1.717 (1.259 to 2.342)	1.865 (1.286 to 2.703)	1.560 (1.087 to 2.239)
Pulmonology							
emergency	1.103 (0.879 to 1.383)	1.035 (0.816 to 1.311)	1	1.028 (0.810 to 1.305)	1.076 (0.846 to 1.368)	1.262 (0.985 to 1.618)	1.208 (0.939 to 1.555)
elective	1.018 (0.704 to 1.473)	0.679 (0.457 to 1.010)	1	0.862 (0.573 to 1.295)	1.104 (0.694 to 1.757)	0.962 (0.426 to 2.177)	0.783 (0.364 to 1.685)
Neurology							
emergency	1.317 (0.835 to 2.079)	0.914 (0.559 to 1.497)	1	1.631 (1.042 to 2.553)	1.432 (0.902 to 2.272)	1.515 (0.945 to 2.429)	1.120 (0.674 to 1.861)
elective	1.417 (0.460 to 4.361)	1.148 (0.405 to 3.257)	1	0.988 (0.292 to 3.346)	0.878 (0.210 to 3.662)	2.474 (0.751 to 8.146)	1.776 (0.416 to 7.580)
Otorhinolaryngology							
elective	1.731e+ 02 (0 to Inf)	0.000 (0 to Inf)	1	0.000 (0 to Inf)	3.641 (0 to Inf)	9.926e+ 27 (0 to Inf)	8.552e+ 14 (0 to Inf)
Pediatrics							
emergency	0.963 (0.000 to Inf)	6.194e+ 07 (0.000 to Inf)	1	8.296e+ 07 (0.000 to Inf)	3.748e+ 07 (0.000 to Inf)	8.155e+ 07 (0.000 to Inf)	3.962e+ 07 (0.000 to Inf)
elective	0.000 (0 to Inf)	6.692e+ 23 (0 to Inf)	1	9.922e+ 16 (0 to Inf)	6.100e+ 18 (0 to Inf)	1.351e+ 58 (0 to Inf)	3.127e+ 21 (0 to Inf)

### Elective admissions

Elective patients had a statistically significant weekend effect in in-hospital mortality for the specialties of surgery (*p* = 0.0011), gynecology and obstetrics (*p* = 0.0167), internal medicine (*p* = 0.0000) and pulmonology (*p* = 0.0074) (Table 2). A significantly higher risk for 30-day mortality was seen among elective surgery (*p* = 0.0022), gynecology and obstetrics (*p* = 0.0005) and internal medicine (*p* = 0.0010) patients at the weekend (Table 3).

### Mortality by year

**Fig. 1:**
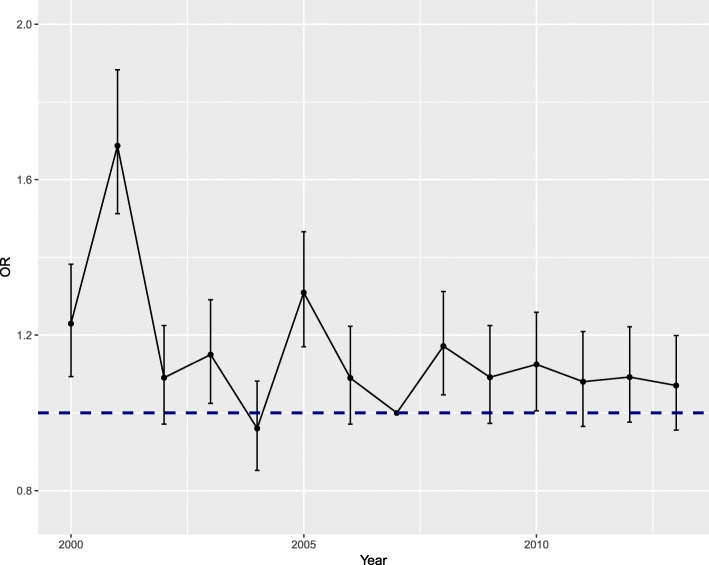
The overall adjusted odds ratio (OR) by year from 2000 to 2013. 2007 is the base line (OR = 1)

**Fig. 2:**
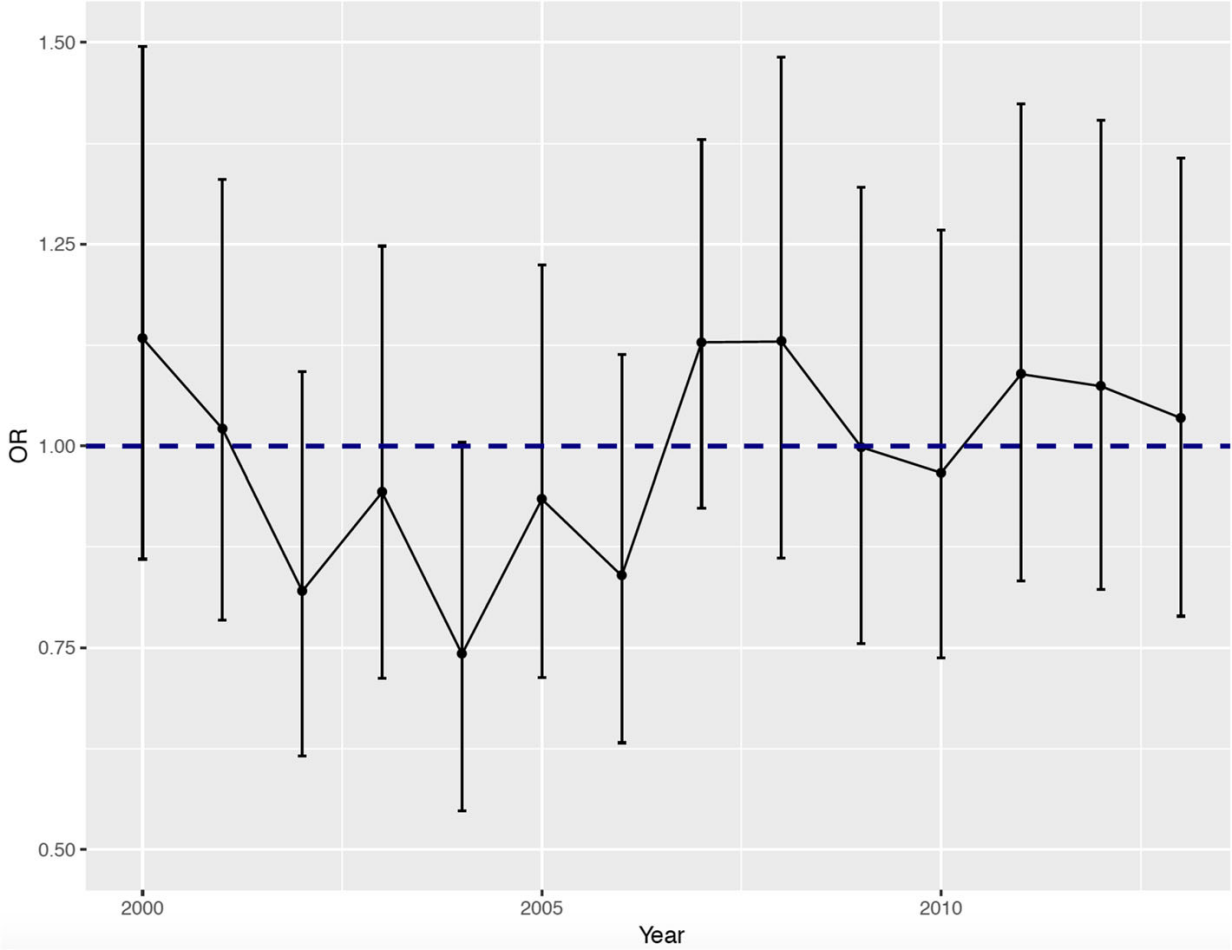
The overall adjusted odds ratio (OR) for weekday admissions (OR = 1) versus weekend admissions by year

The overall adjusted odds ratio of mortality was at its lowest during 2004, with a rather steady rate starting from 2009 and continuing up until 2013 (Fig. 1). The overall adjusted odds of mortality across all specialties during the weekend (Saturday and Sunday) were below weekday mortality for the years 2002–2006 and 2010 (Fig. 2).

### Mortality by sex

Of the specialties with patients of both genders, females had a statistically significant lower risk for in-hospital mortality for emergency acute psychiatry (adjusted OR 0.34, 95% CI 0.120–0.967), emergency surgery (0.80, 0.719–0.895), elective and emergency internal medicine (0.76, 0.624–0.914 and 0.75, 0.706–0.799) and for 30-day post-discharge mortality for emergency and elective acute psychiatry (0.24, 0.127–0.459 and 0.06, 0.008–0.505), emergency surgery (0.84, 0.764–0.916), emergency internal medicine (0.91, 0.856–0.974) and emergency pulmonology (0.73, 0.637–0.836).

### Mortality by specialty

A decline in the annual crude mortality rate was seen for acute psychiatry (0.32% in 2000, 0.28% in 2013), surgery (3.5% in 2000, 2.3% in 2013), gynecology and obstetrics (0.2% in 2000, 0.04% in 2013) and internal medicine (8.4% in 2000, 6.8% in 2013), whereas an increase occurred for pulmonology (6.4% in 2000, 10.1% in 2013), neurology (1.1% in 2000, 5.1% in 2013), pediatrics (0.11% in 2000, 0.14% in 2013) and otorhinolaryngology (0% in 2000, 0.7% in 2013).

## Discussion

We set out to investigate whether the weekend effect existed in the secondary hospitals of our hospital district. For almost all specialties and for both elective and emergency patients, a weekend effect was observed. However, these effects reached statistical significance in only about half of the specialties.

We observe a total of 1170 more deaths at the weekend because of the weekend effect (surgery *n* = 358, internal medicine *n* = 633, pediatrics *n* = 6, gynecology and obstetrics *n* = 19, neurology *n* = 61, acute psychiatry *n* = 4, pulmonology *n* = 89, otorhinolaryngology *n* = 0) when comparing the crude mortality rates of Saturday and Sunday admissions versus Wednesday admissions.

A significant weekend effect was observed in in-hospital and 30-day post-discharge mortality among emergency internal medicine patients. This coincides with previous findings of higher in-hospital mortality for both medical and surgical emergency diagnoses at the weekend [27, 28] and a higher risk for medical interventions [29]. In Finland, internal medicine in a secondary hospital is usually the specialty where medical students start their career. Medical school is a six-year program and fourth-year students are allowed to be on call in internal medicine emergency departments with attending physicians at home available by phone. This is one possible explanation why we only see a significant weekend effect among emergency patients in internal medicine.

For elective patients, a weekend or end-of-week effect was seen for the majority of specialties, which coincides with a current systematic review and meta-analysis [30]. Friday and weekend effects were seen in 30-day mortality among elective surgery patients [2]. The number of patients on Saturdays and Sundays was about one-third to one-half of that during the week, with this difference due in part to fewer elective patients. Fewer elective patients in turn increase the proportion of emergency to elective patients. Previously, some have hypothesized that fewer elective patients at the weekend is one reason for the weekend effect [6]. In our hospital district, sicker elective patients tend be admitted a day or two before surgery for monitoring, which most likely also contributes to the weekend effect seen in elective patients. Elective procedures on the weekend are often performed in order to shorten long wait times. These procedures are performed in addition to a regular 40-h work week. Staff fatigue may be one reason why those admitted at the weekend for an elective procedure had a higher risk of mortality.

### Mortality by specialty and centralization of services

The centralization of healthcare services is a controversial topic. It has been at the forefront of political debate in Finland. Centralization increases the amount of a certain patient type treated, increasing experience through repetition. Conversely, patients may have to be transported a great distance to reach the centralized treatment center, thus falling prey to the golden hour phenomenon [31]. Patients living in the catchment area of Helsinki University Hospital may travel up to 200 km when off-hours specialized care is not available in a closer secondary hospital. Those in need of the advanced specialized treatment only Helsinki provides may travel up to 1200 km from northernmost Lapland.

There were no weekend effects for the specialties of acute psychiatry, otorhinolaryngology, neurology and pediatrics. The number of patients in these specialties was a fraction during the weekend of what it was during the week. The most difficult cases were also most likely transferred to the university hospital, thus leaving the simpler cases to be treated at the secondary hospitals and decreasing the probability of a weekend effect. Emergency otorhinolaryngology patients are centralized completely to the university hospital. Internal medicine was the only specialty with a weekend effect in both in-hospital and 30-day post-discharge mortality for both elective and emergency patients despite certain more serious conditions, for example ST-elevation myocardial infarction, being centralized to the university hospital. The specialties of surgery, internal medicine, and gynecology and obstetrics are the most sensitive to the weekend effect in both the university hospital and secondary hospitals. While the centralization of services to the university hospital is usually justified with the notion of decreased patient mortality, we found more statistically significant weekend effects in the non-centralized specialties at the university hospital than at secondary hospitals [24]. Numerous studies have shown that the centralization of low-volume surgical procedures, oncologic surgery and trauma patients lowers mortality [32–34]. However, this is not true in every diagnosis and patient group [35]. Therefore, we must delve into which diagnoses and patients benefit from centralization, which is the next step in our research.

Recently, weekend effect research was criticized for not scrutinizing care pathways before admission to hospital, with this being the reason for the weekend effect as opposed to a downturn in quality of care at the weekend [36]. Nevertheless, we observed a weekend effect even though our hospital admission pathway is the same on weeknights and at the weekend. In addition, the weekend effect was only visible in certain specialties and not detected in intensive care patients, the sickest patients of all [24].

### Strengths of this study

As all residents of Finland are entitled to necessary health care and no off-hours private hospitals exist, our data is a very accurate depiction of the in-hospital treatment received in the municipalities of the greater Helsinki area. The catchment area of the hospital district remained the same during the whole study. The ancestry of the inhabitants of Finland is rather uniform [37], allowing us a relatively consistent patient set in respect to mortality risks.

### Limitations of this study

Administrative data are noted for a variety of problems due to errors and missing data. We found that some specialties recorded co-morbidities well but others mainly recorded only the diagnoses pertaining directly to the hospital episode in question. In other words, in the case of a broken hip, codes for hip fracture and ensuing pneumonia are recorded but not for example diabetes, asthma and hypertension. Even when diagnosis codes are recorded, they do not tell of the severity of illness or whether the patient has reached their treatment target. A lack of co-morbidities in the data necessitated the use of the abovementioned risk categories to assess illness severity. While some have found a weekend effect regardless of illness severity [38], others showed the effect to be caused by sicker patients at the weekend [39]. Notwithstanding, the effect of disease severity is offset by the magnitude of this study population.

Patients’ socioeconomic variables were also not included. Hospitals in Finland do not record income, race, education level and other socioeconomic factors. This information also cannot be extrapolated from e.g. the patient’s address or zip code. When this study began in 2000, only 1.8% of the inhabitants of Finland were of foreign descent (0.5% of non-Caucasian descent), with only 3.8% of foreign descent (1.1% of non-Caucasian descent) in 2013 when this study finished, creating a rather uniform population [40]. The Finnish government has strived to avoid the development of underprivileged areas and public housing is interspersed all over the city. As education and health care is in practice free, there are not great differences between people or socioeconomic strata. Salaries and progressive income tax have also prevented the forming of the very rich or the very poor. As an example, the mean salary in Finland in 2018 was 3465 euros per month, the mode 2600 euros and median 3079 euros [41]. Due to these reasons, socioeconomic variables were not included.

Due to the lack of reliable time stamps, we were not able to differentiate between admissions occurring late Friday or early Monday morning versus those during office hours. This may be the cause of the end-of-week effect for some specialties as weekend staffing is from Friday 3:30 pm to Monday 8:00 am. The lack of co-morbidities and time stamps may allow for an omitted-variable bias but we attempted to compensate for this with the categorization of illness severity. Public holidays were not calculated as being part of the weekend if they did not fall on the weekend. We chose not to include public holidays as there were few during the week (between seven to ten per year) and this small amount was unlikely to affect our findings radically. The same approach was applied by Mohammed et al. with a similar number of bank holidays per year (eight) [11].

One historical effect was relevant in this study. Two secondary hospitals joined the university hospital in 2001. These two hospitals were included in this study for the year 2000 up until their joining Helsinki University Hospital in 2001. A clear spike is seen in mortality risk by year in 2001, which is possibly due to organizational changes and rearranging of services connected to the merger.

All six secondary hospitals included in the study belong to the hospital district of the same university hospital. They are all on the same level in regard to the severity of patient illness and care provided, as well as number of beds and staffing levels. Their only major difference is each hospital’s geographic catchment area. The hospital district’s catchment area has remained the same for the entire study period. All hospitals are, in practice, teaching hospitals as every doctor in Finland is required to guide and teach those less experienced. While the majority of medical students are trained at the university hospital, they do also receive training in these secondary hospitals. Residents also receive training during their specialization in these secondary hospitals. Due to these reasons, these variables were not included in our analyses. Adjusting for the particular secondary hospital might reduce possible confounding. However, in our opinion, it is improbable that adding the hospital as a random effect would materially change our results.

## Conclusions

Findings of a higher risk for mortality for emergency and elective patients admitted at the weekend necessitate further research into the reasons and solutions for this problem. Solutions, including staffing with fewer residents and more specialists at the weekend, as well as a seven-day-a-week service, have been proposed. Nevertheless, restructuring of the resident versus specialist ratio would most likely require more doctors, thus ruling it infeasible in most countries without major changes in the healthcare system. Internal medicine, surgery, and gynecology and obstetrics were the specialties most sensitive to the weekend effect. We must examine the specific diseases, especially in these three specialties, that are sensitive to the weekend effect in order to focus funds and staffing changes accordingly. This is the next step for our group’s research. The centralization of services to the university hospital does not seem to eliminate the weekend effect, which undermines claims of centralization improving patient safety. Limitation of elective admissions during the weekend is crucial. Application of criteria for weekend elective admissions, e.g. reminiscent of day surgery criteria, is a possible approach to reducing the weekend effect through better patient selection.

## Data Availability

The datasets used and analyzed during the current study are available from the corresponding author on reasonable request.

## Abbreviations

CI: Confidence interval
ICD-10: International Classification of Diseases
km: Kilometer
OR: Odds ratio
